# Supplementary material for: Analyzing the Differential Impact of Semen Preparation Methods on the Outcomes of Assisted Reproductive Techniques
Source: Biomedicines. 2023 Feb 6;11(2):467. doi: 10.3390/biomedicines11020467 (PMC9953211; doi:10.3390/biomedicines11020467)
Supplement: Supplementary file 1 [file biomedicines-11-00467-s001.zip › biomedicines-2151987-supplementary.pdf]

Supplementary Material

Analysing the Differential Impact of Semen Preparation Methods towards the Outcomes of Assisted Reproductive Techniques

Riffat Bibi, Sarwat Jahan, Tayyaba Afsar, Ali Almajwal, Mohamad Eid Hammadeh, Houda Amor ,Ali Abusharha and Suhail Razak

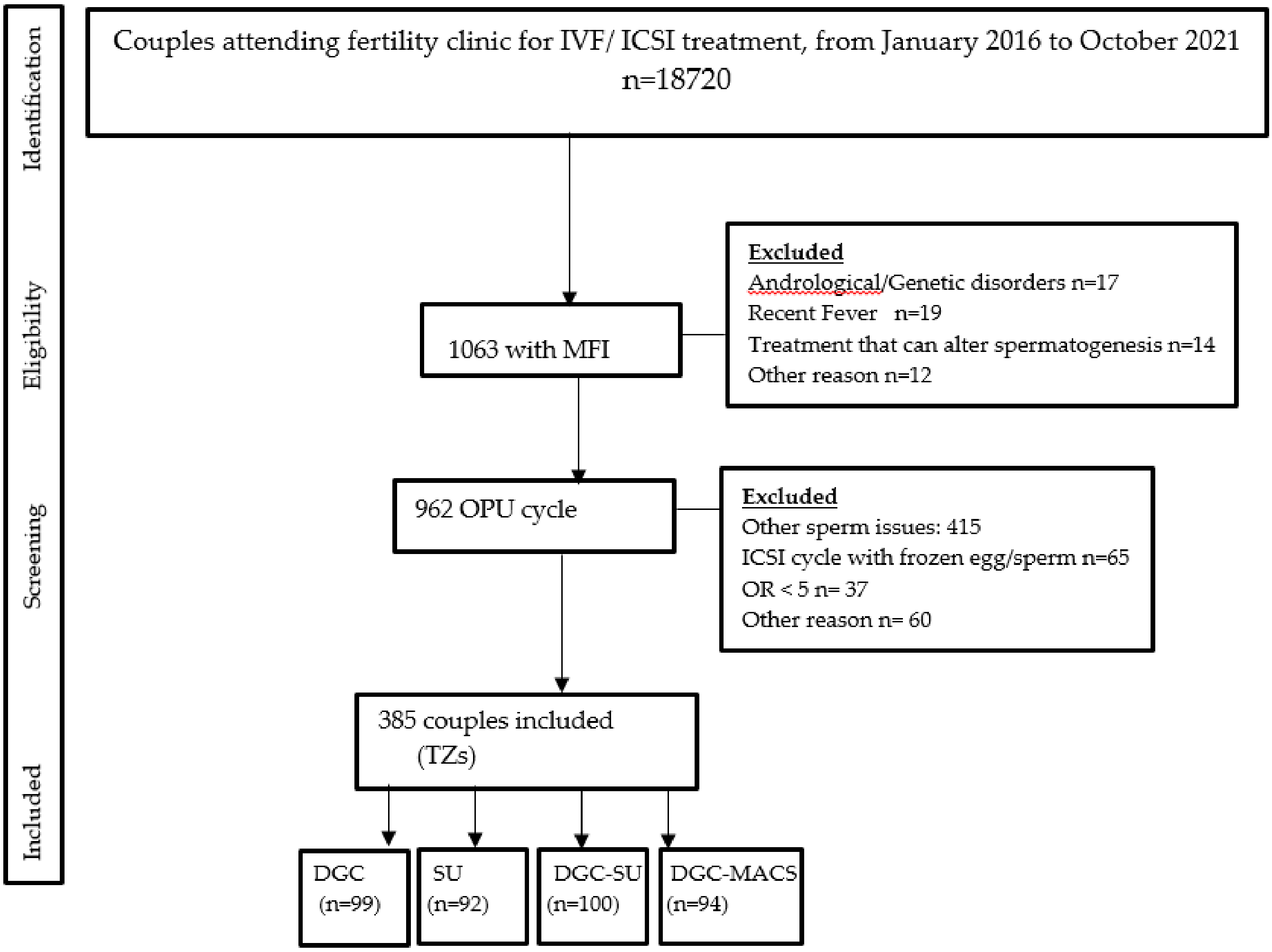

Figure S1. Schematic of the recruitment in the study. OR, oocyte retrieve; OPU, oocyte pick-up; TZs, Teratozoospermia.
DGC, Gradient centrifugation; SU, Swim-up; MACS, magnetically activated cell selection. .
